# Rapid UV-Vis spectrophotometric method aided by firefly-PLS models for the simultaneous quantification of ciprofloxacin, lomefloxacin, and enrofloxacin in their laboratory mixture, dosage forms and water samples: greenness and blueness assessment

**DOI:** 10.1186/s13065-024-01286-0

**Published:** 2024-09-16

**Authors:** Ali Alqahtani, Taha Alqahtani, Adel Al Fatease, Enas H. Tolba

**Affiliations:** 1 Department of Pharmacology, College of Pharmacy, King Khalid University, Abha, 62529 Saudi Arabia; 2 Department of Pharmaceutics, College of Pharmacy, King Khalid University, Abha, 62529 Saudi Arabia; 3 Egyptian Drug Authority (EDA), Giza, 35521 Egypt

**Keywords:** Fluoroquinolone, Spectrophotometric methods, Chemometric models, Greenness evaluation

## Abstract

Herein, a novel UV spectrophotometric method coupled with chemometric tools was developed for the simultaneous determination of three fluoroquinolone antibiotics: ciprofloxacin, lomefloxacin, and enrofloxacin. Such integration of UV spectroscopy and chemometric analysis proved to be a simple, rapid, and cost-effective approach for the quantification of these clinically important pharmaceutical compounds and aid in their quality control analysis. The method employed firefly algorithm for variable selection and partial least squares (PLS) regression for model calibration. The developed method was validated by independent test set in addition the accuracy, intra and inter-day precision as per ICH guidelines which showed a satisfactory performance with mean recovery ranged between 98.18 and 101.83 with %RSD < 2. Besides, the developed method displayed ultrasensitive levels with LODs (0.0803, 0.1125, 0.1309 µg/mL) and LOQs (0.2434, 0.3409, 0.3968 µg/mL) for ciprofloxacin, lomefloxacin, and enrofloxacin, respectively. The greenness and blueness of the developed method were also evaluated using the recently proposed Analytical GREEnness metric approach (AGREE) and Blue applicability grade index (BAGI) tools, which showed a high AGREE score of 0.79 and a BAGI score of 77.5. These results indicate that the developed method provides an environmentally friendly alternative to the traditionally used chromatographic techniques, while maintaining high analytical practicability. Finally, the application of the developed methodology was demonstrated on real pharmaceutical and tap water samples, and the results were in good agreement with those obtained by the reference HPLC method indicating the reliability and suitability of the proposed spectrophotometric method for routine analysis of fluoroquinolone antibiotics.

**Supplementary Information:**

The online version contains supplementary material available at 10.1186/s13065-024-01286-0.

## Introduction

Fluoroquinolones represent a group of synthetic broad-spectrum antibiotics that have been widely utilized in both human and veterinary medicine [1]. Effective against various types of bacteria, they are particularly valuable in addressing different infectious diseases [2]. Among the most frequently used fluoroquinolones, ciprofloxacin, lomefloxacin, and enrofloxacin have demonstrated significant therapeutic benefits [3]. These antibiotics function by hindering bacterial DNA gyrase and topoisomerase IV, which are crucial enzymes for DNA replication and transcription, ultimately resulting in the death of bacterial cells [4]. Nevertheless, their extensive usage has given rise to resistant bacterial strains; hence it is important to carefully consider their use to minimize resistance development [5]. Furthermore, monitoring their presence in food products from animals is vital for ensuring public health safety since these antibiotics can endure in the environment and enter the human food chain [6].

From an analytical perspective, the determination of fluoroquinolone levels requires accurate and sensitive techniques. The literature survey revealed that several analytical techniques have been developed for the quantification of ciprofloxacin, lomefloxacin, and enrofloxacin alone or in combination with other medications or degradation products. These methods include UV spectrophotometry [7–9], spectrofluorimetry [9–15], electro-analytical techniques [16–21], and chromatographic methods such as HPLC [22–25] and LC-MS/MS [26–29]. Regarding their simultaneous determination, only chromatographic methods such as HPLC coupled with fluorescence detection [30] and micellar electrokinetic capillary chromatography [31] have been reported thus far, although these techniques may not be readily available in many quality control laboratories due to their high cost and complexity. In addition, tedious sample preparation procedures and longer analysis times are often encountered with these methods.

UV spectrophotometry represents a relatively simple, rapid, and cost-effective analytical technique that offers good sensitivity for the determination of fluoroquinolone antibiotics. Nevertheless, the quantification of multiple fluoroquinolones in complex matrices can be challenging due to the overlapping of their UV absorption spectra. To address this issue, the application of chemometric tools such as Partial Least Squares (PLS) regression [32, 33] and variable selection algorithms like Firefly Algorithm (FA) [34–36] can be beneficial to resolve spectral interferences and establish predictive models for the simultaneous determination of multiple fluoroquinolone antibiotics. Such a combination of FA and PLS has been demonstrated to be advantageous for the analysis of many pharmaceutical compounds either based on their UV spectral or chromatographic fingerprints [37, 38]. Furthermore, the employment of chemometric techniques either based on regression models or design of experiment approaches (DoE) allows the development of green analytical methods by reducing sample preparation steps and avoiding the use of toxic organic solvents while maintaining good analytical performance [39–43].

The aim of this study is to develop a rapid and eco-friendly analytical method for the simultaneous determination of ciprofloxacin, lomefloxacin, and enrofloxacin in pharmaceutical formulations as well as water samples using UV spectrophotometry. The developed method involves the application of FA for wavelength selection and PLS for multivariate calibration. PLS was chosen for its ability to handle the significant spectral overlap and potential collinearity in UV-Vis data of these compounds. This multivariate technique excels in dimension reduction and noise filtering, crucial for extracting relevant information from complex spectral data. Moreover, PLS allows for simultaneous modeling of multiple analytes, making it ideal for our multi-component analysis. Its robustness against spectral interferences and strong predictive power, especially when coupled with variable selection techniques like the FA, positions PLS as an optimal choice for developing a reliable and accurate quantification method. By utilizing PLS in our approach, we aim to overcome the limitations of traditional univariate methods and achieve precise determination of ciprofloxacin, lomefloxacin, and enrofloxacin in various matrices. Additionally, a comparative assessment has been conducted to evaluate the greenness and environmental friendliness of the developed method compared to conventional chromatographic techniques using the Analytical GREEnness metric approach (AGREE) [44]. Furthermore, a comparative study between the developed method and reported literature has also been undertaken focusing on their practicality through an evaluation with the Blue applicability grade index (BAGI) tool [45] i.e. method blueness, which provides a systematic quantitative approach to assess analytical methods’ real-world applicability.

## Experimental

### Reagents and materials

Pure ciprofloxacin, lomefloxacin, and enrofloxacin reference standards were obtained from the Egyptian Drug Authority (EDA), Cairo, Egypt with certified purities of 99.32%, 98.75%, 99.25%, respectively. Analytical grade acetic acid with purity 99.7% was acquired from El-Nasr Pharmaceutical Chemicals Co. (Cairo, Egypt). Water used throughout this study was double distilled. Pharmaceutical formulations containing ciprofloxacin (Cipro 500 mg/tablet), lomefloxacin (lomeflox 400 mg/tablet) were purchased from local pharmacies (Cairo, Egypt). Byatril chewable tablets containing 68 mg enrofloxacin per tablet were obtained from a veterinary pharmacy (Cairo, Egypt).

### Instrumentation

A Shimadzu UV-1800 double beam spectrophotometer (Kyoto, Japan) equipped with a 1 cm quartz cell was used for all UV absorbance measurements. The acquisition parameters include a wavelength range from 200 to 400 nm, data interval of 1 nm and a fast scan speed and band width of 1 nm. UV probe version 2.43 software was used for data acquisition and processing before chemometric analysis.

### Standard solutions

Stock solutions of ciprofloxacin, lomefloxacin, and enrofloxacin (100 µg/mL each) were prepared separately by dissolving the appropriate amounts of each drug in 10% aqueous acetic acid. Working solutions (20 µg/mL) were then prepared by appropriate dilution of the stock solutions with water to be employed in the spectrophotometric measurements and chemometric analysis.

### Procedures

#### Calibration and validation set constructions

The first critical step in the chemometric optimization of this simultaneous determination method involved the construction of appropriate calibration and validation sets. Brereton fractional factorial design was utilized to generate a training set encompassing 25 synthetic mixtures of the three analytes at varying concentration levels. The choice of the concentration levels was based on their linearity in the UV absorption range as well as maximizing the concentration ranges for each analyte to enable the development of robust multivariate models. The central level of this design corresponded to 4 µg/mL for each antibiotic and the low and high concentration levels were set at 2 and 6 µg/mL, respectively.

An additional 20 validation mixtures were also prepared independently using a central composite design following the same concentration ranges to assess the predictive performance of the developed chemometric models. This design encompasses central, axial and factorial points to ensure a comprehensive validation of the developed method.

#### Chemometric analysis

The UV absorption spectra of the calibration and validation sets were first exported to MATLAB^®^ R2016a environment (MathWorks Inc., Natick, MA, USA) version (9.0.0.341360) for chemometric processing. Initially, regions above 370 nm and below 220 nm were excluded due to weak signals and potential interference, respectively. PLS-1 models were then developed using the entire 220–370 nm spectral range to simultaneously quantify the three analytes. Cross-validation method leaving out one sample at a time was employed to determine the optimum number of latent variables. These optimized models were further incorporated using the FA to select the most significant wavelengths for each analyte. The Firefly Algorithm mimics the social behavior of fireflies to identify the subset of variables that minimize the root mean squared error in prediction. The parameters affecting the behavior of the FA, such as the number of fireflies, maximum generations, absorption coefficient, and randomization parameter, were optimized through combinatorial testing to achieve the best variable selection performance. The absorption coefficient parameter, denoted as “γ”, is one of the most crucial parameters in the optimization process of the Firefly Algorithm. This parameter regulates the light intensity and, consequently, the attractiveness of the fireflies. Its value is pivotal in determining the speed of convergence and the overall behavior of the algorithm. The “α” parameter, on the other hand, provides a random movement to the fireflies. Without this random component, the fireflies may be attracted to a suboptimal light source, leading to a solution restricted to a local optimum. By appropriately selecting the value of this parameter, the search can escape from any local solution, thereby increasing the chances of finding the global optimum. The fitness function in the Firefly Algorithm was set to minimize the root-mean-square error of the PLS models. The selected wavelengths obtained through the Firefly Algorithm were then used to re-build the PLS-1 models for each analyte, and the performance of the optimized models was evaluated using the independent validation set using figures of merit such as relative root mean square error of prediction (RRMSEP) and bias-corrected RRMSEP (BCRRMSEP). Furthermore, the accuracy and precision of the developed method were evaluated as per ICH guidelines through the analysis of different samples in the same day and over 3 consecutive days to assess the intra- and inter-day variability, respectively.

#### Application to pharmaceutical formulations and water samples

The validated FA-PLS chemometric models were applied for the determination of ciprofloxacin, lomefloxacin and enrofloxacin in their tablet dosage forms. To prepare the sample solutions, 5 tablets of each formulation were accurately weighed, finely powdered and an accurately weighed amount of the powdered tablet equivalent to 10 mg of each active ingredient was transferred to a 100 mL volumetric flask and diluted to the mark with 10% acetic acid. The samples were sonicated for 20 min and filtered through a 0.45 μm membrane filter. The filtered solutions were then diluted with water to obtain working solutions labeled to contain 20 µg/mL of each analyte. These sample solutions were further subjected to the optimized FA-PLS procedure for the simultaneous determination of the three fluoroquinolone antibiotics.

The validated FA-PLS models have also been used to determine these analytes in spiked tap water samples, investigating their applicability for analyzing the antibiotics in environmental matrices. Five milliliters of water samples were spiked with working standard solutions of ciprofloxacin, lomefloxacin, and enrofloxacin to achieve final concentrations of 2, 3, 4, and 5 µg/mL. These samples were then analyzed using the developed FA-PLS procedure for the simultaneous determination of these antibiotics without requiring any special sample preparation.

## Results and discussion

### Spectral characteristics

**Fig. 1:**
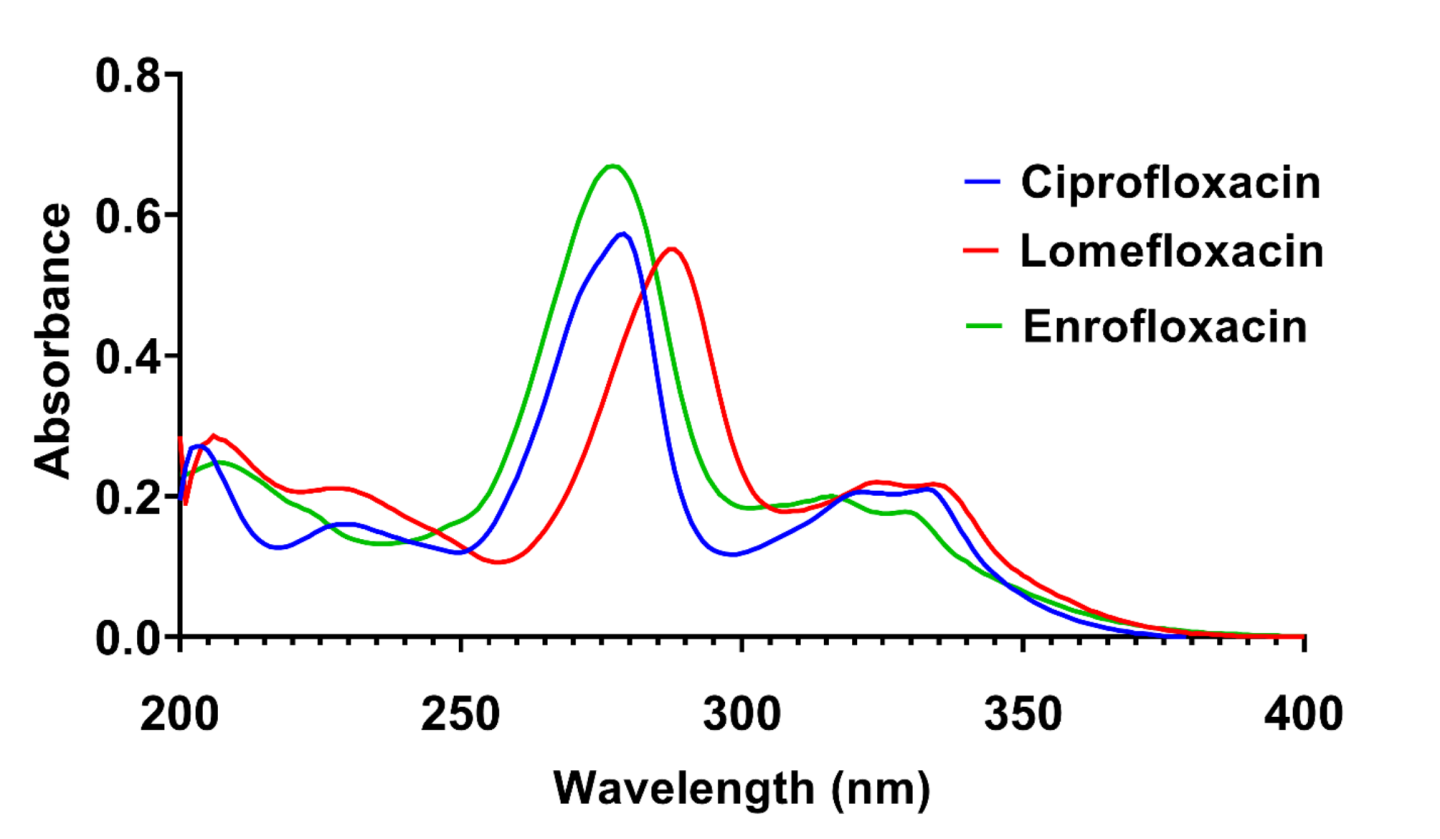
Zero order absorption spectra of (5 µg/mL) of ciprofloxacin, lomefloxacin and enrofloxacin showing sever overlap hindering their direct determination in their ternary mixture

The UV absorption spectra of the three fluoroquinolone antibiotics showed significant overlap in the 220–370 nm range, as shown in Fig. 1. Ciprofloxacin exhibited an absorption maximum at 280 nm, while lomefloxacin and enrofloxacin showed absorption maxima at 288 and 277 nm, respectively. This spectral overlap, combined with the very close structural similarity of the analytes, poses a significant challenge for their simultaneous determination using conventional UV-Vis spectroscopic methods. Therefore, chemometric tools were employed to enhance the selectivity and accuracy of the proposed method. It is worth noting that the choice of diluent system (10% aqueous acetic acid in water) was crucial for optimizing method performance and applicability. This selection was based on several key factors. Firstly, the pH-dependent solubility of fluoroquinolones necessitated an acidic environment to ensure complete dissolution and maintain the drugs in their protonated, more soluble form. The 10% aqueous acetic acid provides this optimal condition. Secondly, this acidic medium contributes to the stability of the fluoroquinolone solutions, critical for maintaining consistent spectral characteristics over time and ensuring the reliability of our chemometric models. Moreover, the use of aqueous acetic acid followed by water dilution aligns with green analytical chemistry principles, avoiding organic solvents and making the method more environmentally friendly and cost-effective compared to HPLC methods.

### Chemometric models development and optimization

**Fig. 2:**
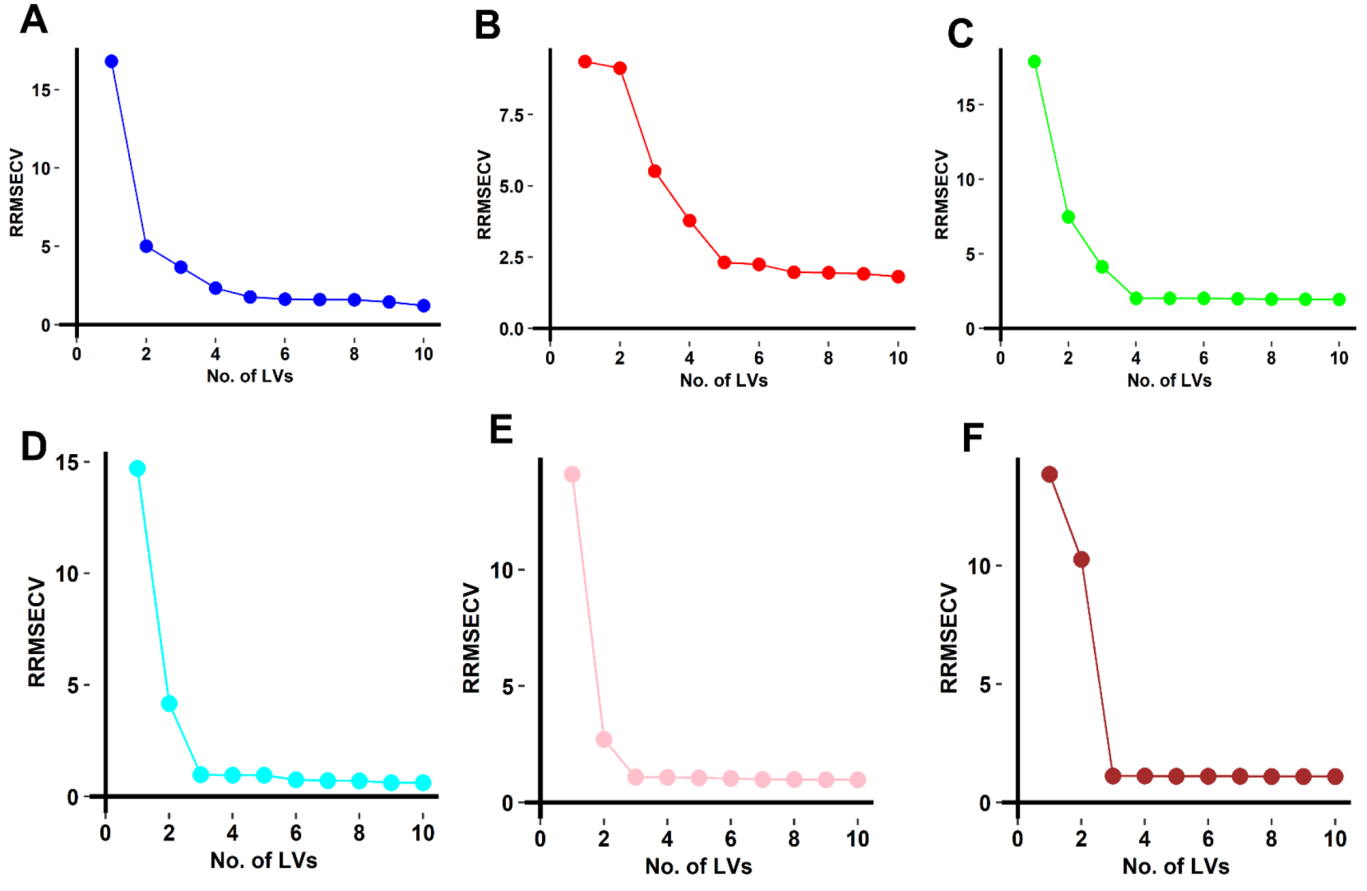
Cross-validation results using leave-one-out procedures of the full PLS-1 models for **(A)** ciprofloxacin, **(B)** lomefloxacin, **(C)** enrofloxacin and and the FA-PLS models for **(D)** ciprofloxacin, **(E)** lomefloxacin and **(F)** enrofloxacin. The optimum number of latent variables for both models shows a significant decrease in their RRMSECV values

Among different supervised multivariate regression models, PLS-1 was chosen for the simultaneous quantification of the three analytes due to its robustness and ability to handle highly correlated spectral data. As an initial step, full-spectrum PLS-1 models were developed using the entire 220–370 nm range of the calibration set (Table S1), which provided reasonable predictive performance for the three analytes. The critical steps in PLS-1 models optimization involves the determination of the optimal number of latent variables and the selection of the most discriminating wavelengths. Cross-validation method leaving out one sample at a time was employed to evaluate the minimum number of latent variables required to capture the maximum variation in the data with sufficient accuracy as calculated using the relative root mean square error of cross-validation (RRMSECV). Results indicate the optimum number of latent variables to be 5 for both ciprofloxacin, lomefloxacin, and 4 for enrofloxacin, respectively as shown in (Fig. 2A-C).

An additional step was undertaken to further enhance the performance of the PLS-1 models by utilizing the FA for variable selection. This algorithm has shown great promise in identifying the most significant wavelength regions for analyte quantification particularly in UV spectral data. The algorithm behaves in two phases the exploration phase where the fireflies explore the entire spectral range randomly to identify potentially significant variables, followed by an exploitation phase where the algorithm refines the variable selection by focusing on the promising regions identified in the previous step. The optimal FA parameters such as the number of fireflies, maximum generations, absorption coefficient, and randomization parameter were determined through grid search experimentation to achieve the best variable selection performance (Table S3). The fitness function was set to minimize the RMSECV as calculated by the PLS-1 models and the variable selection was continued until no further improvement in the RMSECV was observed. The FA led to reducing the original spectral variables from 151 to just 40, 39, and 37 for ciprofloxacin, lomefloxacin, and enrofloxacin, respectively, while maintaining excellent predictive capability. The selected variables for each analyte were incorporated in the final PLS-1 models and interestingly the number of latent variables required also decreased to 3 for all three analytes (Fig. 2D-F) posing the potential for more robust and accurate models.

### Models’ validation

**Fig. 3:**
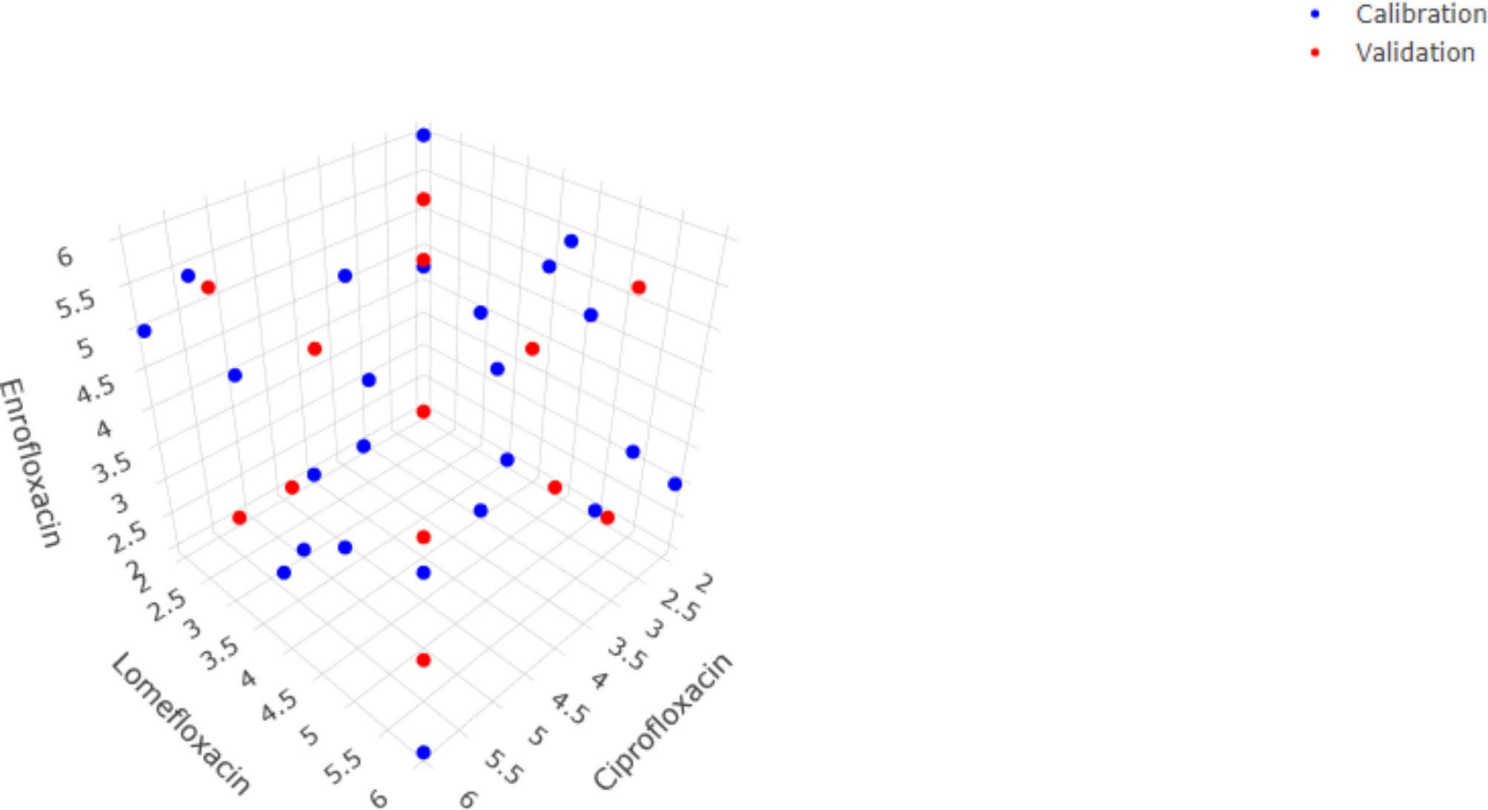
The 3D experimental space showing the positioning of the calibration and validation samples for ciprofloxacin, lomefloxacin and enrofloxacin

Since the FA-PLS models outperformed the full-spectrum PLS models, they were selected for further validation by employing an independent validation set (Table S2) & (Fig. 3). Several validation parameters have been assessed including relative root mean square error of prediction (RRMSEP), bias-corrected RRMSEP and coefficient of determination (R²) for the validation set as shown in Table 1. The developed models displayed excellent agreement between the predicted and reference values for all three analytes with slope values close to 1, intercepts close to 0 and high R2 values above 0.999 which confirms their suitability for the simultaneous quantification of ciprofloxacin, lomefloxacin and enrofloxacin in the external validation set. Analysis of different validation metrics indicated that the FA-PLS models yielded excellent predictive performance with RRMSEP values below 2% for all three analytes (Table 1).

**Table 1:** Validation results of ciprofloxacin, lomefloxacin and enrofloxacin by the proposed FA-PLS models

	Drug	Slope^a^	Intercept^a^	*R* ^2 a^	LOD (µg/mL)	LOQ (µg/mL)	RRMSEC	RRMSEP	RBCMSEP
**FA-PLS**	**Ciprofloxacin**	1.0050	-0.0146	0.9997	0.0803	0.2434	0.6103	1.3393	0.0717
	**Lomefloxacin**	1.0043	0.0026	0.9995	0.1125	0.3409	0.9682	1.2723	0.0647
	**Enrofloxacin**	0.9981	0.0270	0.9992	0.1309	0.3968	1.0840	1.2724	0.0648

^a^ Data obtained by plotting the actual versus the predicted values

In addition to these analytical figures of merit, the validated FA-PLS models were also evaluated for their accuracy and precision as per the ICH guidelines by analyzing different concentrations of the studied analytes in the same day and in three consecutive days. The results summarized in (Table S4) show that the method exhibits excellent accuracy with mean %R ranged between 98.18 and 101.83 and high intraday and interday precision with RSD values below 2% for all analytes suggesting the reliability of the developed UV-Vis spectrophotometric method aided by chemometric tools in the routine analysis of these fluoroquinolones.

The stability of the fluoroquinolones in the chosen diluent system (10% aqueous acetic acid followed by dilution with water) was also evaluated and displayed excellent stability for ciprofloxacin, lomefloxacin, and enrofloxacin. The acidic environment created by acetic acid helps maintain the drugs in their protonated form, which not only enhances solubility but also contributes to their chemical stability. Over a 24-hour period at room temperature, the UV absorbance spectra of the drug solutions remained consistent, with less than 2% variation in peak intensities. This stability was crucial for the reliability of the developed FA-PLS models, as it ensured consistent spectral characteristics throughout the analysis process. Furthermore, when stored at 4 °C, the solutions maintained their spectral integrity for up to 4 days, demonstrating the robustness of the sample preparation approach. The stability provided by this diluent system is particularly advantageous for routine analysis in quality control settings, where sample solutions may need to be prepared in advance or analyzed over extended periods. Additionally, this stability contributes to the method’s overall precision and accuracy, as evidenced by our low RSD values in both intra-day and inter-day analyses (Table S4).

### Greenness and blueness assessment

Initial assessment of the ecological impact of the developed method revealed its ‘greenness’ compared to more traditional liquid chromatography-based approaches [30]. The proposed UV-Vis spectrophotometric method eliminated the need for organic solvents, complex sample preparation steps and lengthy chromatographic runs, thus significantly reducing the environmental footprint of the analytical procedure. Moreover, the number of samples that can be analyzed per hour is much higher compared to HPLC, enhancing the overall throughput and productivity.

**Fig. 4:**
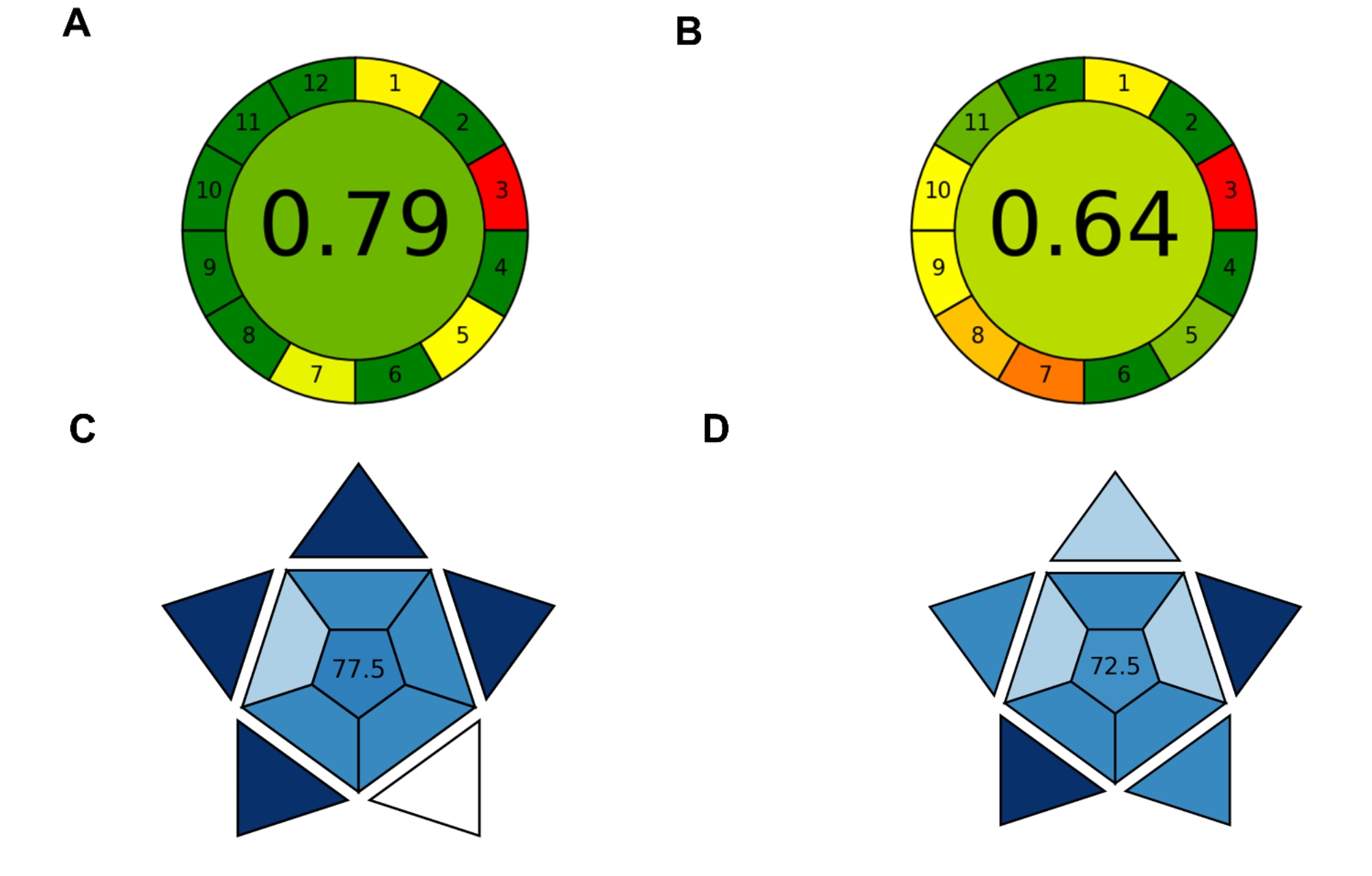
Greenness assessment of **(A)** the developed FA-PLS method and **(B)** the reported chromatographic method using the AGREE tool to determine their environmental impact. Blueness assessment of **(C)** the developed FA-PLS method and **(D)** the reported chromatographic method using the BAGI tool to determine their analytical practicability

A widely used metric to assess the environmental impact of analytical methods is the AGREE metric which takes into account the 12 principles of green chemistry which has been applied in this study. The AGREE analysis indicates that the proposed UV-Vis/chemometrics method has a significantly higher AGREE score of 0.75 compared to the reported HPLC approach [30] which are around 0.65 (Fig. 4). Such results are mainly due to items such as energy consumption, solvent consumption, hazardous waste production, and high throughput that are greatly improved in the developed UV-Vis /chemometrics method.

Another new metric is the BAGI assessment which evaluates the method analytical practicability i.e. blueness in order to ensure the desired analytical performance in a quantitative manner. BAGI evaluation of the developed method yielded a score of 77.5 indicating the high analytical reliability, efficiency and suitability of the proposed UV-Vis/chemometrics method for the simultaneous determination of the three fluoroquinolone antibiotics under investigation (Fig. 4C). This analysis transcended the reported chromatographic method which scored only 72.5 (Fig. 4D) mainly due to shorter analysis time, ease of use, and high throughput characteristics posing the potential of the developed method for routine quality control applications.

### Application to pharmaceutical formulation and water samples

The validated FA-PLS models were successfully applied for the simultaneous quantification of ciprofloxacin, lomefloxacin and enrofloxacin in a commercial pharmaceutical formulation. The models exhibited excellent predictive accuracy with mean recoveries ranging from 98.12 to 101.25% and low RSD values between 0.785 and 1. 402% as shown in (Table 2), confirming the suitability of the developed method for accurate and precise quantification of the studied fluoroquinolones in pharmaceutical samples. Moreover, the results obtained by the proposed UV-Vis spectrophotometric method were in good agreement with those obtained by the reference HPLC methods [22, 25] in terms of mean and variance, demonstrating the reliability and validity of the proposed green and blue analytical protocol. Furthermore, the applicability of the proposed method was extended to the determination of the studied fluoroquinolones in tap water samples. Spiked samples showed satisfactory recovery values ranging from 98 to 102% with low standard deviations, demonstrating the feasibility of the developed FA-PLS models for environmental monitoring of theses analytes (Table 3).

**Table 2:** Quantitative analysis of ciprofloxacin, lomefloxacin and enrofloxacin in commercial pharmaceutical preparations by the proposed methods and statistical comparison with the reported methods

Drug	Method	Mean^a^	SD	Variance	t-test (2.306)^b^	*P* value	F-value (6.338)^b^	*P* value
**Ciprofloxacin**	FA-PLS	99.33	1.517	2.302	1.423	0.194	1.578	0.669
	Reported method [25]	100.88	1.906	3.632				
**Lomefloxacin**	FA-PLS	100.15	2.054	4.221	0.085	0.935	1.843	0.568
	Reported method [22]	100.05	1.513	2.29				
**Enrofloxacin**	FA-PLS	99.88	1.022	1.045	0.445	0.67	2.437	0.41
	Reported method [25]	100.26	1.596	2.547				

^a^ Average of five determinations

^b^ The values in parenthesis are tabulated values of “*t* “and “*F*”

**Table 3:** Quantitative analysis of ciprofloxacin, lomefloxacin and enrofloxacin in tap water samples using the developed FA-PLS models

Amount added (µg/mL)	Ciprofloxacin Recovery (%) ± SD ^a^	Lomefloxacin Recovery (%) ± SD ^a^	Enrofloxacin Recovery (%) ± SD ^a^
3	99.15 ± 0.546	101.76 ± 1.19	100.21 ± 0.956
4	101.15 ± 0.342	98.18 ± 0.993	99.83 ± 1.009
5	99.64 ± 0.628	100.11 ± 0.941	101.83 ± 0.844
6	101.53 ± 1.255	101.57 ± 1.294	99.81 ± 0.894

^a^ Average three measurements

## Conclusion and future directions

The present study evaluates the application of a UV-Vis spectrophotometric method combined with chemometric tools for the simultaneous determination of three fluoroquinolone antibiotics, namely ciprofloxacin, lomefloxacin and enrofloxacin. PLS coupled with FA as variable selection procedure allowed the development of accurate, precise and robust quantification models with a significant reduction in the number of spectral variables. The developed FA-PLS models showed excellent performance in terms of accuracy and precision and were successfully applied for the determination of the studied analytes in an independent validation set. The method also displayed improved greenness and blueness compared to the existing HPLC-based methods, proving its suitability for routine quality control applications in pharmaceutical laboratories as well as environmental water samples.

While our current study successfully developed a green UV-spectrophotometric method for the simultaneous determination of ciprofloxacin, lomefloxacin, and enrofloxacin, there are additional avenues for investigation, particularly considering the photosensitive and fluorescent properties of fluoroquinolones. Future studies could benefit from incorporating spectrofluorometric analysis, which may provide complementary and potentially more sensitive results, especially in studying the photolysis of these compounds. Such an approach could offer valuable insights into the photodegradation behavior of these antibiotics, potentially enhancing the sensitivity of the analytical method. Furthermore, spectrofluorometric analysis could be instrumental in exploring any fluorescence-based interactions between these compounds in mixed samples. These considerations open up new directions, potentially leading to even more comprehensive analytical methods for fluoroquinolones in various matrices.

## Electronic supplementary material

Below is the link to the electronic supplementary material.

Supplementary Material 1

## Data Availability

The data presented in this study are available on request from the corresponding author.
