# Supplementary material for: Rapid UV-Vis spectrophotometric method aided by firefly-PLS models for the simultaneous quantification of ciprofloxacin, lomefloxacin, and enrofloxacin in their laboratory mixture, dosage forms and water samples: greenness and blueness assessment
Source: BMC Chem. 2024 Sep 16;18(1):172. doi: 10.1186/s13065-024-01286-0 (PMC11406862; doi:10.1186/s13065-024-01286-0)
Supplement: Supplementary file 1 — Supplementary Material 1 [file 13065_2024_1286_MOESM1_ESM.docx]

**Rapid UV-Vis Spectrophotometric Method Aided by Firefly-PLS Models for the Simultaneous Quantification of Ciprofloxacin, Lomefloxacin, and Enrofloxacin in their Laboratory Mixture, Dosage Forms and Water Samples: Greenness and Blueness Assessment**

**Ali Alqahtani ^a^, Taha Alqahtani ^a^, Adel Al Fatease ^b^, Enas H. Tolba ^c, *^**

^a^ Department of Pharmacology, College of Pharmacy, King Khalid University, Abha, 62529, Saudi Arabia

^b^ Department of Pharmaceutics, College of Pharmacy, King Khalid University, Abha, 62529, Saudi Arabia

^c^ Egyptian Drug Authority (EDA), Giza, 35521, Egypt

*Corresponding author email address **(Enas H. Tolba**): [nosso2@yahoo.com](mailto:nosso2@yahoo.com)

**Table S1:** The concentrations of the calibration set as calculated *via* implementing a partial factorial experimental design approach.

| No. | Ciprofloxacin (µg/mL) | Lomefloxacin (µg/mL) | Enrofloxacin (µg/mL) |
| --- | --- | --- | --- |
| 1 | 4 | 4 | 4 |
| 2 | 4 | 2 | 2 |
| 3 | 2 | 2 | 6 |
| 4 | 2 | 6 | 3 |
| 5 | 6 | 3 | 6 |
| 6 | 3 | 6 | 4 |
| 7 | 6 | 4 | 3 |
| 8 | 4 | 3 | 3 |
| 9 | 3 | 3 | 5 |
| 10 | 3 | 5 | 6 |
| 11 | 5 | 6 | 5 |
| 12 | 6 | 5 | 4 |
| 13 | 5 | 4 | 6 |
| 14 | 4 | 6 | 6 |
| 15 | 6 | 6 | 2 |
| 16 | 6 | 2 | 5 |
| 17 | 2 | 5 | 2 |
| 18 | 5 | 2 | 4 |
| 19 | 2 | 4 | 5 |
| 20 | 4 | 5 | 5 |
| 21 | 5 | 5 | 3 |
| 22 | 5 | 3 | 2 |
| 23 | 3 | 2 | 3 |
| 24 | 2 | 3 | 4 |
| 25 | 3 | 4 | 2 |

**Table S2:** The concentrations of the validation set as calculated *via* implementing a central composite experimental design approach with 5 central points.

| No. | Ciprofloxacin (µg/mL) | Lomefloxacin (µg/mL) | Enrofloxacin (µg/mL) |
| --- | --- | --- | --- |
| 1 | 5.5 | 2.5 | 2.5 |
| 2 | 2.5 | 5.5 | 2.5 |
| 3 | 2.5 | 2.5 | 2.5 |
| 4 | 2.5 | 5.5 | 5.5 |
| 5 | 4 | 4 | 4 |
| 6 | 5.5 | 5.5 | 2.5 |
| 7 | 5.5 | 5.5 | 5.5 |
| 8 | 4 | 4 | 4 |
| 9 | 4 | 4 | 4 |
| 10 | 5.5 | 2.5 | 5.5 |
| 11 | 2.5 | 2.5 | 5.5 |
| 12 | 4 | 4 | 4 |
| 13 | 4 | 4 | 4 |
| 14 | 4 | 4 | 4 |
| 15 | 4 | 5.8 | 4 |
| 16 | 4 | 2.2 | 4 |
| 17 | 5.8 | 4 | 4 |
| 18 | 4 | 4 | 2.2 |
| 19 | 2.2 | 4 | 4 |
| 20 | 4 | 4 | 5.8 |

**Table S3:** The optimized parameters of the Firefly algorithm as a variable selection procedure to enhance the PLS models' predictability.

| Parameter | Ciprofloxacin | Lomefloxacin | Enrofloxacin |
| --- | --- | --- | --- |
| Number of fireflies | 44 | 48 | 40 |
| Maximum generations | 300 | | |
| α | 0.2 | 0.3 | 0.1 |
| β_ο_ | 1.2 | 1.5 | 1 |
| γ | 1 | | |

**Table S4:** Accuracy and precision results for the determination of ciprofloxacin, lomefloxacin and enrofloxacin by the proposed FA-PLS method.

| *Concentration (ng/mL)* | | | *Accuracy*  *(% R ± SD)* [*^a^*](https://www.sciencedirect.com/science/article/pii/S1386142524003305#tblfn1) | | | | *Precision (%RSD)* [*^a^*](https://www.sciencedirect.com/science/article/pii/S1386142524003305#tblfn1) | | | | | |
| --- | --- | --- | --- | --- | --- | --- | --- | --- | --- | --- | --- | --- |
| Ciprofloxacin (µg/mL) | Lomefloxacin (µg/mL) | Enrofloxacin (µg/mL) | Ciprofloxacin | Lomefloxacin | Enrofloxacin | Ciprofloxacin | | | Lomefloxacin | | Enrofloxacin | |
|  |  |  |  |  |  | *Intra-day* | | *Inter-day* | *Intra-day* | *Inter-day* | *Intra-day* | *Inter-day* |
| 2 | 3 | 4 | 99.15  ±  1.257 | 101.76  ±  0.403 | 100.21  ±  1.190 | 1.078 | | 1.93 | 0.821 | 0.147 | 0.698 | 0.705 |
| 3 | 4 | 5 | 101.15  ±  0.753 | 98.18  ±  1.200 | 99.83  ±  0.993 | 0.862 | | 1.814 | 0.879 | 1.008 | 0.754 | 0.54 |
| 4 | 2 | 2 | 99.64  ±  0.978 | 100.11  ±  0.546 | 101.83  ±  0.941 | 0.805 | | 1.412 | 0.698 | 1.541 | 0.418 | 0.371 |
| 5 | 5 | 3 | 101.53  ±  0.873 | 101.57  ±  0.342 | 99.81  ±  1.294 | 1.194 | | 1.611 | 0.754 | 0.511 | 0.262 | 0.888 |

[^a^](https://www.sciencedirect.com/science/article/pii/S1386142524003305#tblfn1) Average of three determinations
